# Standardizing TEER Measurements in Blood-Brain Barrier-on-Chip Systems: A Systematic Review of Electrode Designs and Configurations

**DOI:** 10.3390/biomimetics11020119

**Published:** 2026-02-05

**Authors:** Nazanin Ghane, Reza Jafari, Naser Valipour Motlagh

**Affiliations:** Department of Applied Sciences, University of Quebec in Chicoutimi, Chicoutimi, QC G7H 2B1, Canada; naser.valipour-motlagh1@uqac.ca

**Keywords:** blood-brain barrier-on-chip, dynamic BBB models, electrodes, organ-on-chip, static BBB models, transendothelial electrical resistance

## Abstract

The blood-brain barrier (BBB) is one of the most selective physiological interfaces in the human body. Transendothelial electrical resistance (TEER) has become a widely adopted quantitative metric for assessing its in vitro structural and functional integrity. Although TEER measurements are routinely incorporated into BBB-on-chips, the absence of harmonized electrode architectures, measurement settings, and reporting standards continues to undermine reproducibility and translational reliability among laboratories. This systematic review provides the first comprehensive classification and critical comparison of electrode configurations used for TEER assessment, specifically within BBB-on-chip systems. Eligible studies were analyzed and categorized according to electrode design, fabrication method, integration strategy, and operational constraints. We critically evaluated six principal electrode architectures, highlighting their performance trade-offs in terms of uniformity of current distribution, long-term stability, scalability, and compatibility with dynamic shear conditions. Furthermore, we propose a bioinspired TEER reporting framework that consolidates essential metadata, including electrode specification, temperature control, viscosity effects, and blank resistance correction. Our analysis proposes screen-printed and hybrid silver-indium tin oxide (ITO) electrodes as promising candidates for next-generation BBB platforms. Moreover, our review provides a structured roadmap for standardizing TEER electrode design and reporting practices to facilitate interlaboratory consistency and accelerate the adoption of BBB-on-chip systems as truly biomimetic platforms for predictive neuropharmacological workflows.

## 1. Introduction

The central nervous system (CNS), comprising the brain and spinal cord, relies on the blood-brain barrier (BBB) to maintain its delicate and highly regulated microenvironment. This dynamic interface manages nutrient transport, ion balance, and immune-CNS communication and minimizes CNS toxin exposure. The dysfunction and dysregulation of the BBB often have severe consequences and are linked to multiple sclerosis, Alzheimer’s disease, Parkinson’s disease, stroke, and traumatic brain injury [1]. Because the CNS has almost no natural regenerative capacity, treating such disorders depends on advanced delivery strategies, including targeted carriers, cell therapies, and theranostics [2,3,4]. Yet despite decades of work, fewer than 2% of small molecules and essentially no large neurotherapeutics can cross an intact BBB [5], which partly explains why only around 7% of CNS drugs in development ever reach market [1].

The structure and functioning of the BBB serve as a fundamental template for biomimetic microphysiological system design. Traditional 2D in vitro BBB models, such as Transwell systems, remain useful for basic drug screening because they are simple and inexpensive [6,7]; however, their static culture environment fails to mimic the physiological forces and complexity of the living barrier. Animal models, long considered the preclinical gold standard, often do not translate into human outcomes, with over 80% of promising drug candidates failing during trials [8,9]. In response to these challenges, microfluidic BBB-on-chip technologies have emerged as a powerful alternative. By enabling co-culture under controlled flow and integrating biosensors in real-time, noninvasive monitoring, they are a closer analogy to the human neurovascular environment [10,11]. Nonetheless, even as BBB-on-chip technologies advance, transendothelial electric resistance (TEER) measurement strategies used to track biomimetic fidelity remain fragmented. However, a lack of standardized, reproducible electrode design continues to limit both mechanistic research and translational applications.

Despite the increasing adoption of TEER in BBB-on-chip research, prior reviews have largely focused on methodological and physicochemical factors affecting TEER measurements in conventional BBB models. For example, the comprehensive analysis by Vigh et al. [12] critically evaluated how the variables of temperature, medium conductivity, membrane characteristics, and current distribution affect TEER results. However, such reviews treat TEER primarily as a measurement result and do not systematically examine how electrode architecture, integration strategy, and geometry govern the measurement fidelity, reproducibility, and translational relevance of BBB-on-chip systems. Moreover, existing TEER-related reviews that address impedance measurement techniques in BBB models have focused primarily on the electrical sensing methodology and the associated biophysical parameters rather than on systematic classifications of electrode architectures. For example, Vigh et al. [12] critically reviewed TEER measurement methodologies across BBB models, emphasizing the effects of electrode placement, current density, frequency dependence, and medium conductivity on impedance-based techniques. However, such reviews have not offered a systematic overview of a design-based classification of electrode architectures specific to BBB-on-chip models and do not comparatively evaluate electrode integration strategies under static vs. dynamic microfluidic conditions.

To address this gap, the present work for the first time introduces a PRISMA-based, design-centered classification of TEER electrode configurations and map their geometry, fabrication strategies, current distribution characteristics, and applicability to static vs. dynamic microfluidic environments. Moreover, this review proposes a TEER reporting framework that consolidates essential metadata to support interlaboratory comparability. Together, these contributions establish a novel perspective in which TEER is interpreted not merely as a biological metric but as an engineered function of electrode design itself.

## 2. Search Strategy and Study Selection

A systematic search strategy was implemented to ensure comprehensive and reproducible coverage of the literature related to BBB-on-chip platforms and TEER measurements with a particular focus on electrode design and integration. Searches were conducted in PubMed (using “Title/Abstract field”), Scopus (using “TITLE-ABS-KEY”), and Google Scholar (top 300 results) for publications available up to 18 August 2025. The core search terms were defined a priori and combined using Boolean operators as follows: (“blood-brain barrier” OR BBB OR “blood brain barrier”) AND (“on-chip” OR “organ-on-chip” OR “organ on chip” OR microfluidic OR microfluidics) AND (“transendothelial electrical resistance” OR TEER OR “electrical resistance”) AND (electrode OR “impedance spectroscopy” OR impedance). Only English publications were screened. All thesis/dissertation and conference abstracts without full text (as gray literature) were excluded. Then, the retrieved records were imported into EndNote, and duplicates were removed. The selection process was conducted in two steps:

1. Title and abstract screening by two independent reviewers;
2. Full-text assessment for inclusion or exclusion.

The inclusion criteria included: experimental or review studies related to BBB-on-chip and TEER measurements using electrodes; quantitative TEER data (Ω·cm^2^ or spectra); and a clear description of measurement methods. Moreover, the exclusion criteria included studies limited to Transwell models with no microfluidic context and theoretical papers without experimental methodology.

To ensure comparability across studies, all extracted TEER values of tissue (R) based on Ohm’s law method were normalized to Ω·cm^2^ according to the following equation [13]:

(1) $$R=\left(Rt-Rb\right)\times A,$$

where Rb is the blank resistance of the membrane (without cells), and Rt is the total resistance of the sample containing cultured cells, normalized for culture area (A). Moreover, measurement settings were standardized following minimum information for reporting TEER in BBB-on-chip system recommendations and measurements at 37 °C. For each record, electrode geometry, spacing, and measurement temperature were recorded whenever available. Normalized TEER values were categorized according to flow condition (static or dynamic), electrode type, geometry/design, fabrication method, and cell source (primary, immortalized, or induced pluripotent stem cell (iPSC)-derived) for subsequent analysis.

Finally, a quality assessment was performed on the basis of five key criteria: complete device description, TEER measurement methodology, environmental control, reproducibility and statistics, and comparison with standard references. Studies were classified as “high,” “moderate,” or “low” quality. In this regard, each study was evaluated using a quantitative scoring framework with a maximum score of 20. The assessment criteria included electrode accuracy, long-term stability, scalability, fabrication cost, clean-room dependency, and suitability for dynamic microfluidic conditions, as summarized in Table 1. The total score was then used to classify studies as “high” (≥15), “moderate” (10–14), or “low” (<10).

### PRISMA Flowchart and Screening Process

**Figure 1:**
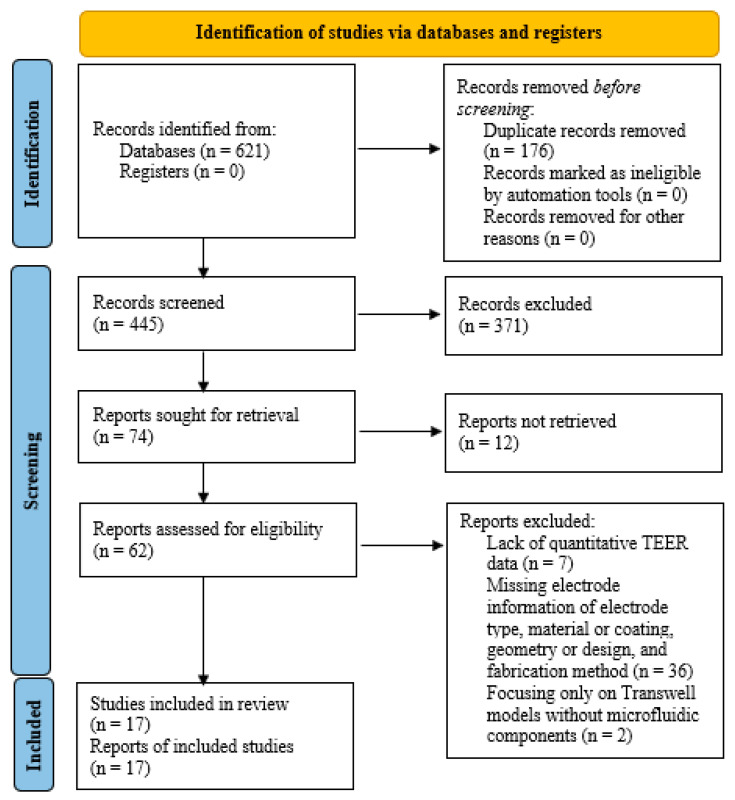
PRISMA 2020 flow diagram showing the identification, screening, eligibility, and inclusion stages for BBB-on-chip studies focused on TEER and electrode architecture.

The systematic review process followed PRISMA 2020 guidelines to ensure transparency and reproducibility. Figure 1 summarizes the article identification, screening, eligibility assessment, and inclusion stages.

A total of 621 records were initially found from the PubMed, Scopus, and Google Scholar databases. After removing 176 duplicates, 445 unique studies remained for title and abstract screening. Based on relevance to BBB-on-chip and TEER measurements, 62 records were selected for text evaluation. During the eligibility assessment, 45 articles were excluded due to the lack of quantitative TEER data, missing electrode information, or focusing only on Transwell models without microfluidic components. A substantial proportion were excluded because they lacked quantitative TEER data (*n* = 7), did not report sufficient electrode-specific information, such as electrode type, material or coating, geometry or design, or fabrication method (*n* = 36), or focused exclusively on conventional Transwell models without microfluidic components (*n* = 2). Finally, 17 studies met the inclusion criteria and were incorporated into the qualitative synthesis.

## 3. Overview of BBB Structure and Functions

The BBB is essential for maintaining the tightly controlled homeostasis of the CNS, as it regulates the selective exchange of ions, metabolites, and biomolecules between the brain parenchyma and the systemic circulation. Hence, mimicking its structure and functions is therefore fundamental for designing in vitro platforms that can truly reproduce or simulate human neurovascular physiology. This section outlines the BBB’s key structural and functional elements, emphasizing how they underpin its unique selectivity and dynamic response to physiological cues.

### 3.1. BBB Features and Functions

The BBB is a semipermeable membranous complex structure found at the blood-CNS tissue interface. This dynamic cellular complex involves tight junctions of endothelial cells formed by junctional adhesion molecules and transmembrane proteins, including occludin and claudin. Hence, a unique and relatively low endocytic activity is created which is formed only in the CNS of the body [14,15]. This structure is mainly responsible for protecting CNS neurons by controlling the environment and the exchange of ions, molecules, and cells. This system allows specific substances to penetrate and maintain the homeostasis of neuronal functions and protects CNS against toxic agents [14]. The functions of the BBB include preventing the paracellular diffusion of hydrophilic substances, transporting nutrients to CNS, transporting hydrophobic molecules and drugs from the brain to blood through efflux pumps, transporting lipophilic agents via transcellular pathways, and regulating the transendothelial migration of blood cells and pathogens [14,15].

### 3.2. Cellular Components of the BBB

**Figure 2:**
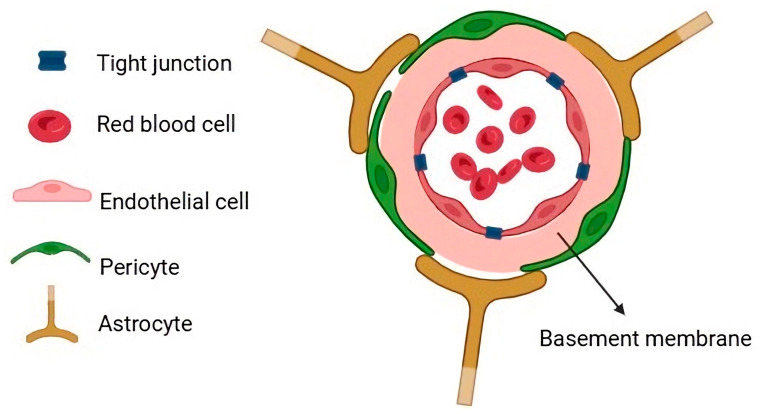
Schematic illustration of the cross-sectional structure of the BBB and its components.

The BBB, as a selective physiological barrier, separates the unique anatomical properties of brain microvascular cells from the blood vessels in the periphery (Figure 2). This structure is composed mainly of three cell types: brain microvascular endothelial cells (ECs), astrocytes, and pericytes.

ECs generally possess both adherent junctions and tight junctions; however, in CNS tissues, they are particularly rich in tight junctions. This structure plays an essential role in tissue integrity and vascular non-permeability and impedes the paracellular transport of a wide range of water-soluble substances, such as polar drugs, and restricts the penetration of small ions. Conversely, lipophilic components can be diffused across the BBB more effectively because of the lipid membrane of the endothelium. Tight junctions consist of transmembrane proteins, including claudin and occludin, that are anchored to the cytoskeleton through scaffolding proteins, including cingulin, zonula occludens-1 (ZO-1), ZO-2, and ZO-3 [16]. It has been reported that a greater number of tight junctions enhances barrier tightness; this feature can be characterized by TEER measurements and permeability coefficients [17]. Shear stress associated with blood flow can be upregulated, attributed to junctional proteins and transporters of the ECs [18]. Hence, fabricating a model with flow-induced shear stress could mimic the native tissues more accurately than traditional in vitro systems, which lack this shear stress [16]. Astrocytes with a star-shaped structure form a continuous sheath that covers the surface of cerebral capillaries; they work as potassium channels to help maintain water homeostasis and ion regulation [16,18]. Moreover, they play a role in the uptake and release of neurotransmitters [19]. Additionally, pericytes wrap around the vessel wall with heterogeneous patterns in the CNS; they have a prominent round nucleus, which differentiates them from the elongated nucleus of the ECs [16]. Pericytes act as phagocytes and clear foreign molecules, maintaining barrier stability and BBB integrity [20].

## 4. BBB Models

A precise representation of the BBB is essential for decoding its transport mechanisms and for accelerating neurotherapeutic discovery. Accordingly, BBB modeling has become a central objective for neuroscientists, pharmaceutical developers, and bioengineers [7]. Although in vivo models can provide accurate therapeutic results, their limitations in regard to quantifying and parameterizing the studies, ethical concerns, and time-consuming and complex procedures cannot be ignored [21].

In vitro models, which are cellular-based systems, enable a precise control of the BBB microenvironment by culturing ECs, pericytes, and astrocytes under defined conditions [7]. In vitro models for BBB mimicking first appeared in the early 1990s [22] as a potential tool to promote human studies and clinical research. These models have numerous advantages, including being cost-effective and versatile, having a manipulable environment, reducing the use of animal models, and demonstrating a greater translatability to human tissues [22,23]. The latest in vitro BBB models can represent complex cellular co-cultures, leading to further precision in simulating physiological human BBB [24]. In some in vitro models, stem cells, including cell types derived from iPSCs, are used to address concerns regarding the reproducibility and availability of human cells. Moreover, these models offer the potential to create isogenic systems derived from the same patient [23]. Recently, several in vitro BBB models have been studied, and they can be classified either as static or dynamic systems. The following section discusses in vitro BBB models, their advantages and disadvantages.

### 4.1. Static In Vitro BBB Models

Static BBB models consist of mono- or multilayer cultures established on plates, flasks, or Transwell inserts. Their simplicity, low cost, and reproducibility make them a standard tool for permeability and transport assays [25]. The Transwell insert uses a microporous membrane (0.4–3 µm pores) separating the luminal and abluminal chambers. Smaller pores are suitable for molecular diffusion studies, whereas immune-cell transmigration assays require pores ≥ 3 µm [25]. Monocultures of ECs on Transwell inserts generally produce low TEER values because of incomplete tight junction formation and weak transporter expression. Co-culture and triple-culture arrangements markedly enhance barrier integrity. For example, Gaillard et al. [26] reported TEER values of 92, 134, and 386 Ω.cm^2^ for EC monolayers, non-contact, and contact co-cultures, respectively. Another study recorded <100, 262, and 350 Ω.cm^2^ for mono-culture, co-culture, and triple-culture rat brain ECs, pericytes, and astrocytes [27]. These data confirm that cell-cell interactions are indispensable for achieving physiologically relevant resistance.

**Figure 3:**
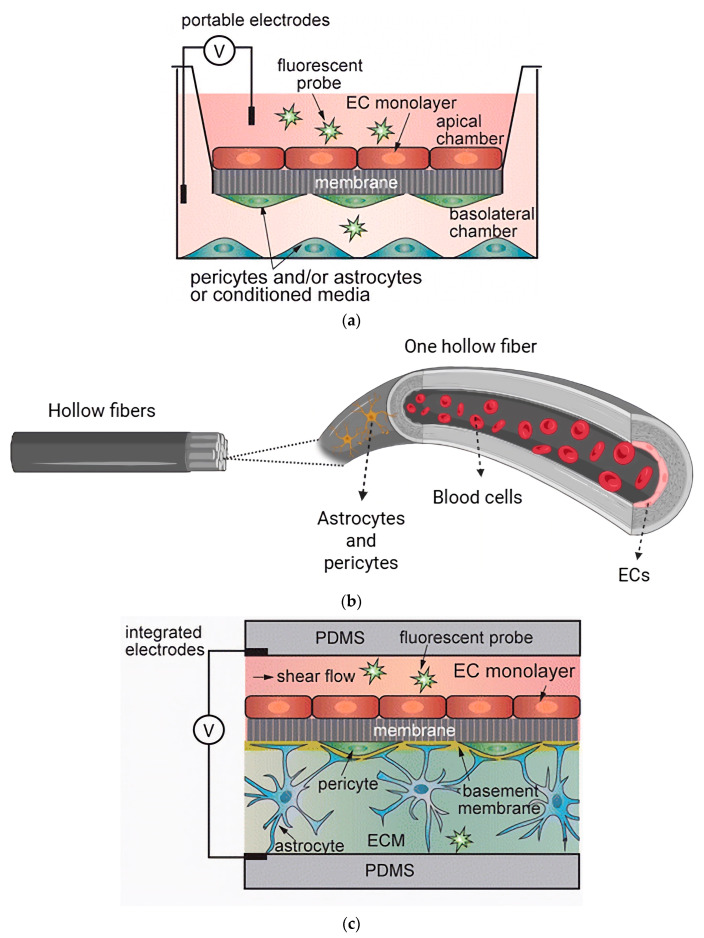
(**a**) Schematic illustration of the Transwell model with an EC monolayer on the apical side of the membrane and astrocyte/pericyte cells in the basolateral chamber, adopted from [54], under the terms of the Creative Commons Attribution 4.0 International License. (**b**) Schematic illustration of the hollow fiber model. (**c**) Schematic illustration of the microfluidic BBB-on-chip model, adopted from [54], under the terms of the Creative Commons Attribution 4.0 International License.

Nevertheless, static models fail to reproduce hemodynamic shear stress, a key determinant of endothelial phenotype and junctional organization [1]. The absence of mechanical cues leads to underdeveloped tight junctions and limited translational accuracy. Consequently, static systems are best suited for mechanistic or screening assays where simplicity outweighs physiological precision. Figure 3a illustrates a schematic of the Transwell model.

### 4.2. Dynamic In Vitro BBB Models

Dynamic models incorporate controlled flow to simulate in vivo shear forces, thereby improving endothelial morphology, transporter activity, and TEER performance [21,28]. In these models, shear stresses of near 6 dyne.cm^−2^, typical of cerebral capillaries, upregulate ZO-1 and other junctional proteins [29,30], and a viscosity adjustment using 3.5% dextran or 6% hydroxyethyl starch (HES) can reproduce blood-like rheology (2.5–4 mPa.s) and stabilize flow patterns [31,32]. Two main dynamic architectures are used: hollow-fiber systems and microfluidic BBB-on-chips.

#### 4.2.1. Hollow Fiber Models

Hollow fiber bioreactors use porous polypropylene fibers coated with extracellular matrix proteins to mimic microvessel geometry [12]. ECs are seeded on the inner lumen and perfused with culture medium, and glial cells reside on the outer surface. Integrated electrodes permit real-time TEER measurements between luminal and abluminal compartments [28]. This configuration successfully reproduces physiological shear stress and stable electrical resistance but limits optical access, which hinders direct imaging and high-throughput analysis and substantially restricts its practical use [33]. Figure 3b shows a schematic illustration of the hollow fiber model.

#### 4.2.2. Microfluidic BBB-on-Chip Models

Microfluidic BBB-on-chip platforms aim to biomimetically recreate physiological cues in the native BBB. They extend dynamic modeling by embedding brain-like microvessel channels within polymeric chips [34]. In general, two microchannels, vascular and parenchymal, are separated by a porous membrane that acts as an artificial basement membrane [35]. Common membrane materials are composed of polyethylene terephthalate (PET) [31,36,37,38,39,40,41,42], polycarbonate (PC) [43,44,45], and polydimethylsiloxane (PDMS) [46,47,48]; each introduces distinct mechanical, optical, and permeability features that influence device performance. Hybrid devices often reinstall PET membranes from Transwell inserts to reduce fabrication complexity while ensuring biocompatibility and consistent porosity [40,41,49,50]. PDMS is attractive for chip fabrication because of its optical clarity, flexibility, gas permeability, and low cost [51,52]. Moreover, it facilitates electrode integration for noninvasive TEER monitoring [33]. Figure 3c shows a schematic illustration of the microfluidic BBB-on-chips.

The relevant studies on BBB-on-chip models are presented in Table 2. Several notable designs have refined these systems. Park et al. [31] developed a PDMS-based chip incorporating a 0.4 µm PET membrane that was coated with collagen IV and fibronectin and seeded with iPSC-derived human brain ECs, astrocytes, and pericytes. Under 3.5% dextran-induced viscosity and 6 dyne.cm^−2^ shear stress, tight junctions and efflux-pump expression increased markedly. Zakharova et al. [46] engineered an eight-channel PDMS chip with a 2 µm PDMS membrane to enable simultaneous testing under uniform flow and demonstrate selective permeability consistent with native BBB function. Salman et al. [34] introduced an open-channel design, which allowed direct gas exchange and high-resolution live imaging to improve nutrient diffusion and visualization. Taking a different approach, Yu et al. [53] proposed a pump-free, gravity-driven PDMS chip that achieved physiological flow rates (50–100 µL.h^−1^), simplified operations, and reduced mechanical noise. PET membranes offer mechanical strength and reproducibility but limit gas exchange, whereas PDMS layers enhance flexibility and imaging yet risk small-molecule absorption. Moreover, iPSC-derived ECs deliver physiological advances but increase costs and have issues with reproducibility, whereas primary human cells provide translational accuracy with limited availability.

Overall, microfluidic BBB-on-chip platforms, as biomimetic models in engineered BBB systems, exhibit TEER values of an order higher than static Transwell models. For instance, Booth and Kim [43] reported TEER levels of 250 Ω.cm^2^ compared to 25 Ω.cm^2^ after three days of culture, attributing this improvement to sustained shear stress and continuous nutrient exchange. Despite this advantage, dynamic models still suffer from fabrication complexity, high costs, and scalability barriers that limit their routine use in drug screening. The continued integration of standardized TEER electrodes and simplified microfabrication may bridge this gap between precision and practicality.

Although dynamic BBB-on-chip models offer superior physiological relevance by incorporating shear stress and continuous perfusion, static BBB systems remain appropriate in some experimental contexts. Static models provide high reproducibility, lower costs, and experimental simplicity, making them particularly suitable for the early-stage optimization of electrode architectures and TEER measurement protocols. In the absence of flow-induced mechanical variables, static systems provide a more controlled assessment of electrode calibration and blank resistance for standardizing TEER measurements.

**Table 2:** Summary of BBB-on-chip models: materials, fabrication methods, membrane types, and cultured cells used in various studies.

Device Architecture			Biological Configuration		Membrane Characteristics		
Device	Chip Materials	Fabrication Method	Surface Coating	Cultured Cells	Membrane Type	Pore Size/Thickness	Ref.
Lab-on-chip	PDMS	Spin-coating and lasers-patterning	Fibronectin	ECs, and astrocytes	PC	0.4 µm/10 µm	[43]
Organ-on-chip	PDMS	Molding	Collagen I	Human cardiac microvascular endothelial cells (hCMECs)/D3	PC	0.4 µm/10 µm	[44]
Organ-on-chip	PDMS	Soft lithography	Fibronectin and collagen IV	Cortical brain microvascular ECs, astrocytes, pericytes	PET	0.4 µm	[37]
Organ-on-chip	Not mentioned	A two and three-lane OrganoPlate	Collagen I	Human brain microvascular endothelial cells (hBMECs), hB astrocytes, hB pericytes	Phase guide	-	[55]
Microfluidic model	PDMS	Purchased from SynVivo, Inc.	Collagen I, human fibronectin, and laminin	hCMECs/D3, astrocytes	-	-	[56]
Organ-on-chip	PDMS	Soft lithography	Collagen IV and fibronectin	iPS-Brain microvascular endothelial cells (BMVECs), astrocytes, pericytes	PET	0.4 µm	[31]
Organ-on-chip	PDMS	Soft lithography	Fibronectin	hCMECs/D3, astrocytes	PET	3 µm	[38]
Organ-on-chip	PDMS	Soft lithography	Collagen I	ECs, astrocytes, pericytes	-	-	[53]
Organ-on-chip	PDMS	A two-step photolithography technique	-	hCMECs/D3, astrocytes, pericytes, human glioblastoma	-	-	[57]
Organ-on-chip	Not mentioned	Donated by AIM Biotech	Fibrin hydrogels	Human umbilical vein ECs, human astrocytes	-	-	[58]
Organ-on-chip	PDMS	Soft lithography	Collagen I	hCMECs/D3, human astrocytes	PDMS	5 µm through-hole pores/~2 µm	[46]
Organ-on-chip	PDMS	Microelectromechanical processes and soft lithography	Fibronectin	ECs, astrocytes	PET	-	[41]
Organ-on-chip	PDMS	Soft lithography	Collagen I	Human brain derived microvascular ECs	-	-	[34]
Organ-on-chip	PDMS	Soft lithography	Collagen I	hBMECs	-	-	[59]
Dual Channel Microfluidics	Biocompatible photoresist containing bisphenol-A-glycidyl dimethacrylate, ethoxylated bisphenol-A-dimethacrylate, carboxyethyl acrylate, PETA, biotinylated resin, and photo initiator (2,4,6–trimethylbenzoyl)–phenylphosphineoxide	3D multiphoton lithography	Gelatin and fibrinogen	Immortalized human umbilical vein ECs, pericytes	PET	-/30 µm	[42]
Organ-on-chip	PDMS	Not mentioned	Collagen IV, fibronectin, laminin	BMECs, astrocytes, pericytes, microglia	PDMS	Multiple pores of 7 µm/50 µm	[47]
Vertical design of a microfluidic chip	PDMS	Not mentioned	Poly-l-lysine, collagen I, hyaluronic acid	NSCs, brain ECs and brain PCs	-	-	[60]
Lab-on-chip	Not mentioned	Not mentioned	Matrigel, gelatin	hEC, pericytes	Polyester	-	[61]
Organ-on-chip	PDMS	Soft lithography and replica molding methods	-	hBMECs, astrocytes, pericytes	-	-	[62]
Organ-on-chip	PDMS	Stereolithography 3D printer and soft lithography	-	BMECs and h pericytes	-	-	[63]
Organ-on-chip	PDMS	Any cubic Photon LCD printer and soft lithography	-	hBMECs	Polyester	0.4 µm	[49]
Organ-on-chip	PDMS	Soft lithography	Collagen and fibronectin	iPSC-derived BMECs, primary pericytes, astrocytes	Permeable PDMS	-	[48]
Organ-on-chip	PDMS	Soft lithography	Injected collagen	hBMECs, hBV pericytes, human astrocytes	-	-	[64]
Organ-on-chip	PDMS	Soft lithography	Fibrin	hiPS-ECs, human brain pericytes, astrocytes	-	-	[65]
Microfluidic model	PDMS	Photomasks and molding	Fibrinogen solution	Human iPS-ECs or HBMECs, astrocytes, pericytes	-	-	[66]
Organ-on-chip	PDMS	Soft lithography	Fibronectin	Human umbilical vein endothelial cells (hUVECs), human astrocytes	Porous Transwell wall immersed in ethylene-glycol-terminated octadecanethiol	-	[50]
Organ-on-chip	PDMS	Lamination photolithography technique and soft lithography	Collagen and fibronectin	hBMVECs, human brain vascular pericytes (hBVPs), astrocytes	PET	-	[40]
Organ-on-chip	PDMS	Soft lithography	Collagen I	hCMECs/D3, human astrocytes, microglial cells	PET porous film	-	[36]
Organ-on-chip	PDMS	Soft lithography	Collagen	hECs, astrocytes, pericytes	-	-	[67]
Organ-on-chip	PDMS	Purchased from Sylgard 184, Dow Corning Corp., Midland, MI	Collagen I	hCMECs/D3, human astrocytes, h pericytes	Semipermeable PET	0.4 µm/12 µm	[39]
Organ-on-chip	PDMS	Soft lithography	Collagen	hCMECs/D3, astrocytes, two breast cancer cell lines	PC	5 µm	[45]
Organ-on-chip	Not mentioned	Purchased an OrganoPlate	Collagen I	hUVECs	-	-	[68]

## 5. BBB-on-Chip Assays

Comprehensive evaluation of BBB-on-chip platforms requires both qualitative and quantitative assays to validate barrier integrity and function [16]. These assessments provide insights into the effects of biochemical and mechanical stimuli on the neurovascular unit and allow comparison between different chip architectures and flow regimes. The most commonly used analytical tools are tight junction immunolabeling, permeability assays, and TEER measurements, which are complemented by cell viability tests, such as live/dead staining [67].

### 5.1. Tight Junction Immunolabeling

**Figure 4:**
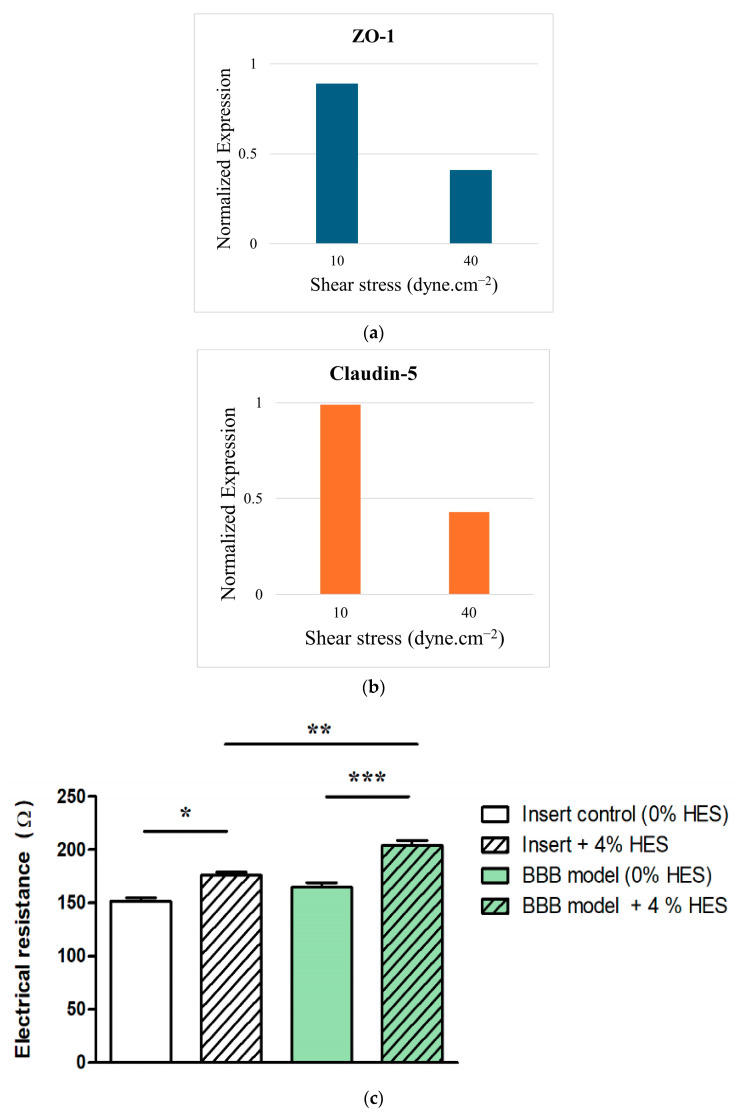
(**a**,**b**) Densitometric analysis of (**a**) ZO-1 and (**b**) Claudin-5 expression in HBMECs shows a significant upregulation at 10 dyne.cm^−2^ relative to 40 dyne.cm^−2^, recreated based on data/information from [69]. (**c**) Increasing TEER of immortalized mouse cerebellar capillary EC (cerebEND) by increasing viscosity after HES treatment (4%); transparent columns: cell culture inserts without cells; green columns: cell culture inserts with the cerebEND cell-line modeling the BBB, two-way ANOVA with Bonferroni post-test; * *p* < 0.05, ** *p* < 0.01, *** *p* < 0.001, adopted from [12], under the terms and conditions of the Creative Commons Attribution (CC BY) license.

Tight junctions, primarily composed of occludins, claudins, and zonula occludens (ZO-1, ZO-2, ZO-3), are the structural foundation of BBB selectivity [1,36]. Their visualization via immunofluorescence or Western blot provides a qualitative index of barrier organization and a means to compare models under different flow conditions [16,43,67]. Dynamic flow environments consistently upregulate tight junction protein expression compared with static cultures. For instance, Garcia-Polite et al. [69] quantified the effect of shear stress on ZO-1 and Claudin-5 expression. ZO-1 levels increased 1.7-fold under a 10 dyne.cm^−2^ flow relative to static conditions, whereas Claudin-5 expression rose up to 2.4-fold. However, excessive shear stress (>40 dyne.cm^−2^) disrupted endothelial morphology, indicating a physiological threshold for flow-induced enhancement (Figure 4a,b). Similarly, Noorani et al. [48] applied immunofluorescence imaging to evaluate ZO-1 and Claudin-5 in induced BMEC-based BBB-on-chips exposed to pulsatile flow (0.15–3 dyne.cm^−2^). Their findings showed uniform endothelial coverage and improved junctional alignment under moderate shear conditions, confirming that physiological pulsation supports tight junction maturation and monolayer stability.

### 5.2. Permeability Assays

Paracellular permeability is a functional marker of BBB integrity that complements structural assays. The use of fluorescent tracers, such as fluorescein isothiocyanate (FITC)-dextran or sodium fluorescein, allows the molecular flux across the barrier (independent of cell viability) to be quantified [16,67,70]. In two-channel systems, the permeability coefficient (P) is derived from the change in tracer concentration over time, as expressed by Campisi et al. [71]:

(2) $$P=\frac{1}{\left(IEt1-ICt1\right)}\times \frac{\left(ICt2-ICt1\right)}{\Delta t}\times \frac{VT}{A}\;,$$

where IE*t*1 is the fluorescence intensity of the medium at the initial time in the endothelial channel. IC*t*1 and IC*t*2 are the fluorescence intensity at initial time and at final time in the other channel, respectively. Δ*t* is the difference between the times, VT is the volume of the other channel, and A is the surface area of the endothelial channel.

Dynamic flow markedly decreases paracellular permeability by reinforcing tight junction assembly. Santa-Maria et al. [61] showed that shear stress reduced the permeability of Lucifer Yellow (LY) and Evans Blue-albumin (EBA) tracers by 78% and 93%, respectively, relative to static conditions, confirming that integrating physiological flow is essential not only for reproducing native BBB morphology but also for functional transport fidelity.

### 5.3. TEER Measurements

TEER quantifies the electrical tightness of endothelial layers and is the gold standard for evaluating BBB integrity [12,41,72]. In a BBB-on-chip device, the TEER value of tissue (R), based on Ohm’s law method, is calculated by the Rb of the membrane (without cells) and Rt of the sample containing cultured cells, all normalized for culture area, as shown in Equation (1) [13].

In impedance spectroscopy-based BBB models, electrical impedance is first measured across a range of frequencies, and TEER is subsequently extracted from the resistive component. Electrical impedance is calculated as the ratio of the voltage/time function to the current/time function, allowing for a more detailed analysis of barrier properties across a range of frequencies. In this method, the parameter of electrical impedance (Z) is applied according to Equations (3) and (4) [13]:

(3) $$Z=\frac{V(t)}{I(t)}=\frac{V0\sin\theta }{I0\sin\left(2\pi ft+Ø\right)}=\frac{1}{Y}\;and$$

(4) Z=ZR+jZI

where V(*t*) and I(*t*) represent the voltage/time function and current/time function, respectively. Moreover, V0, I0, *t*, and *f* are the peak voltage, peak current, time, and frequency, respectively. The parameter Φ is the phase shift between the voltage/time and current/time functions. Y represents the complex conductance, whereas ZR and jZI are the real and the imaginary parts of Z, respectively.

Despite its wide use, TEER analysis remains highly sensitive to experimental parameters, which can confound interpretation. Temperature is the dominant factor, as ion mobility in electrolytes increases exponentially with thermal shifts. Given the linear relationship between conductivity and ion mobility, even minor deviations from physiological temperature (approx. 37 °C) can cause substantial variability in resistance, thereby compromising reproducibility [12,73]. This underscores the need for precise thermal control to generate reliable TEER data. Shear stress, which critically shapes barrier integrity, is another critical determinant of TEER performance. Static BBB cultures, devoid of flow, consistently show lower TEER, whereas dynamic systems promote endothelial alignment, tight junction reinforcement, and transporter regulation [33]. Comparative analyses indicate that BBB-on-chip platforms under physiological flow can achieve TEER values an order of magnitude greater than static Transwell models, demonstrating the functional importance of mechanical cues in barrier maturation [10]. Furthermore, medium viscosity modulates the ionic microenvironment and TEER interpretation. In static cultures with near-saline viscosity (approx. 1 mPa.s), resistance primarily reflects thermal effects, whereas in perfused systems supplemented with viscosity enhancers, such as dextran or hydroxyethyl starch (3–4 mPa.s), elevated osmotic pressure and flow resistance augment shear stress. Despite this mechanistically increasing TEER, it also introduces analytical complexity, requiring careful consideration of viscosity-mediated contributions to barrier function (Figure 4c) [12].

In BBB-on-chip devices, electrode material selection and electrode-cell distance affect long-term endothelial health and measurement reliability. Electrochemical reactions at the electrode-electrolyte interface can produce ionic byproducts and induce localized variations in pH or redox conditions, especially during prolonged TEER monitoring [74,75]. These effects depend on applied materials. Electrochemically inert materials, such as Au, Pt, and ITO, are consistently favored in dynamic BBB-on-chip devices because of their high corrosion resistance, minimal ion dissolution, and higher compatibility with ECs and tight junction integrity. In contrast, more reactive materials, such as Ag and Al, may cause oxidation or ion release in physiological media, which can disrupt the local microenvironment and induce endothelial stress. Ag/AgCl electrodes are nonpolarizable and lower in cost, but they can cause surface degradation, with subsequent signal drift and cytotoxicity effects [74]. An alternative is the use of Pt, but this kind of electrode causes significant resistance at the electrode-medium interface and leads to signal instability [74]. Electrodes positioned too close to the endothelial layer can create localized electric field gradients and amplify electrochemical byproduct accumulation, which can increase the risk of membrane depolarization or junctional disruption [76].

Membrane physicochemical properties, including material composition, thickness, pore size, and pore density, may also influence TEER values such that variations in these parameters can change access resistance. For instance, higher membrane porosity can decrease TEER values [12]. Moreover, microchannel geometry can alter TEER measurements by shaping electric field distribution and the effective sensing volume, such that small channels in microfluidic platforms increase the electrode polarization, and generate a nonuniform electrical current distribution because of the high electrical resistance along the microchannels [77].

Finally, electrode design fundamentally determines measurement fidelity. Electrodes must generate a uniform electric field throughout the microchannel to ensure accurate area-normalized resistance readings [78]. Parameters of geometry, spacing, and electrode material (including platinum (Pt), silver/silver chloride (Ag/AgCl), gold (Au), or indium tin oxide (ITO)) critically influence signal uniformity and noise levels [67,75,79]. Optimized microelectrode architectures are therefore crucial for achieving a consistent and high-resolution TEER characterization in BBB-on-chips.

To promote reproducibility and transparency, we propose a minimal information checklist for reporting TEER data in BBB-on-chip studies, termed the minimum information for reporting TEER in BBB-on-chip systems. Each publication should include at least the following parameters as metadata:

- Geometry and configuration of electrodes: Type (chopstick, interdigitated electrode, etc.), material (Ag/AgCl, Pt, Au, ITO, etc.), dimensions, spacing, and distance between electrode and membrane;
- Environmental control: Measurement temperature, medium, and flow conditions (static or dynamic with rate or shear stress);
- Data normalization: Report both raw resistance (Ω) and normalized TEER (Ω.cm^2^) using Equation (1);
- Replication and statistics: Biological and technical replicates, dispersion (SD or SE), and number of devices (*n*);
- Calibration and blank correction: Electrode calibration date, blank medium resistance, and stability tests;
- Cell and membrane metadata: Cell origin (primary, immortalized, iPSC-derived), co-culture type, membrane material, and coating.

## 6. Structures of TEER Electrodes

Accurate measurement of TEER in BBB-on-chip systems depends critically on electrode architecture and placement [12,67,75]. Over the past decade, a range of electrode designs have been developed to accommodate diverse chip geometries and measurement requirements [43,44,80,81,82,83,84,85,86]. These designs generally fall into two main categories: chopstick electrodes and integrated electrodes, which differ in geometry, material composition, and the degree of coupling with the culture surface. Table 3 summarizes the different types of applied electrodes for TEER measurement in BBB-on-chip devices. Each type has its advantages and drawbacks, which are investigated in the following sections.

Depending on the measurement approach, electrodes in BBB-on-chip platforms are designed either for direct TEER measurements under DC conditions or for impedance spectroscopy, in which frequency-dependent impedance spectra are first recorded and TEER is subsequently extracted as a derived parameter from the resistive component of the low-frequency range.

### 6.1. Chopstick Electrodes

**Figure 5:**
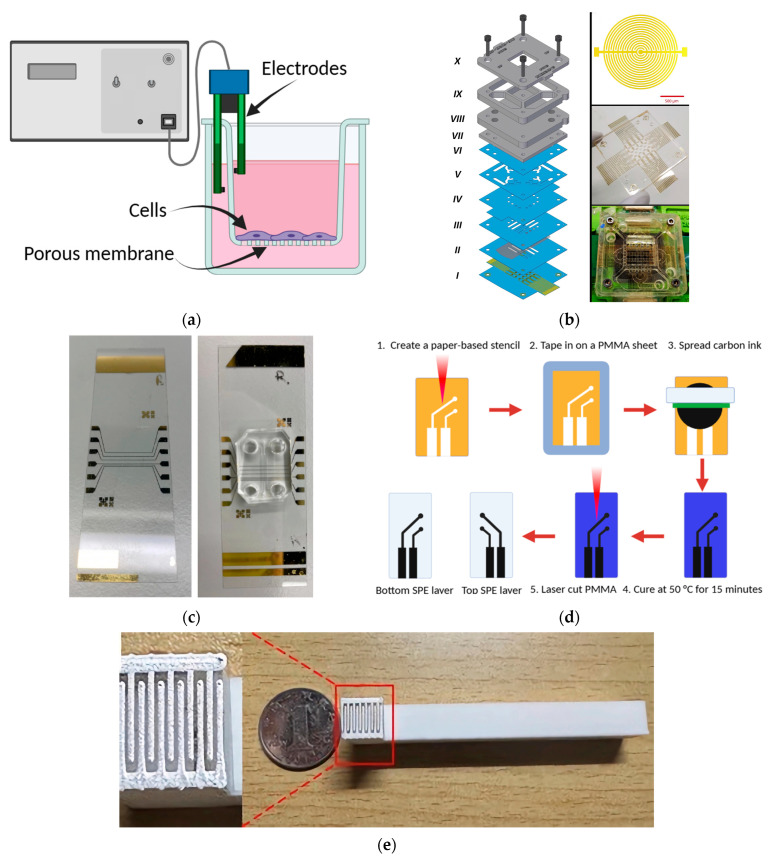
(**a**) Schematic illustration of a wire-based chopstick electrode, figure based on [99]. (**b**) Exploded diagram of an array of concentric interdigitated electrodes (left), and the photograph of the fabricated electrodes (right), adopted from [82], under CC BY-NC-ND 4.0 license. (**c**) BBB-on-chip integrated with a deposited Au electrode, adopted from [67], under a Creative Commons Attribution 4.0 International License. (**d**) Fabrication process and characterization of the carbon-based screen-printed electrode, redrawn based on [98]. (**e**) Photograph of the external controllable electrode of a dynamic Ag-ITO hybrid platform, adopted from [86], under CC BY 4.0 License.

Chopstick electrodes remain the most conventional and accessible tools for TEER measurement in both Transwell and microfluidic BBB models (Figure 5a) [12]. These wire-based probes, typically fabricated from Pt or Ag/AgCl, are manually inserted into opposite fluidic channels or reservoirs, allowing for rapid, parallel measurements without complex instrumentation [10,12]. Their simplicity, affordability, and commercial availability make them a practical choice for standardization studies and early-stage device testing.

Early work by Griep et al. [44] produced a PDMS-based BBB-on-chip equipped with Pt wire electrodes positioned in dedicated grooves. The TEER values measured on-chip (approx. 37 Ω.cm^2^) exceeded those of an equivalent Transwell model (approx. 28 Ω.cm^2^), confirming the improved tight junction integrity achievable in microfluidic systems. Similarly, Kim et al. [87] used a dual-electrode arrangement consisting of a Pt current-carrying electrode and an Ag/AgCl voltage-sensing electrode within a PDMS-PC hybrid chip to achieve TEER values of near 200 Ω.cm^2^ after five days of culturing murine brain ECs. Recent innovations have extended this approach to dynamic and humanized models. Li et al. [88] integrated paired Pt wire electrodes adjacent to endothelial microchannels within a pulsatile-flow BBB-on-chip. This configuration enabled continuous impedance monitoring during the co-culture of hCMECs/D3 cells, pericytes, and astrocytes, and revealed transient barrier rearrangements during lumen formation. Given their chemical inertness, low polarization, and biocompatibility, Pt wire electrodes remain well suited for long-term TEER acquisition in physiologically relevant environments.

However, the chopstick configuration presents several inherent limitations. The most significant challenge is the nonuniform current distribution across the culture area, particularly when the electrode spacing is large relative to the active tissue region [75]. This nonuniformity leads to signal distortion, especially at low TEER values, where the measurement becomes sensitive to electrode positioning and fluid conductivity. At higher resistances and low frequencies (approx. 12.5 Hz), the signal-to-noise ratio decreases sharply, causing impedance spectra to be dominated by thermal and capacitive noise [82]. Another critical drawback is environmental instability. Because these measurements are typically performed outside the incubator, temperature variations can significantly alter ionic mobility and resistance. To address this issue, electrodes must be used with an external heating stage maintaining 37 °C [12]. Hence, these constraints limit chopstick electrodes to qualitative or comparative assessments, rather than high-precision, real-time TEER monitoring.

### 6.2. Integrated Electrodes

In microfluidic BBB-on-chip systems, integrated electrode architectures have been developed to overcome the spatial and electrical limitations of conventional chopstick probes. When symmetrically positioned on both sides of the cellular barrier (typically along the apical and basolateral microchannels), these electrodes establish a homogeneous electric field and uniform current density across the entire culture area, thereby minimizing edge effects and measurement artifacts [41]. This configuration allows the electrode geometry to be precisely scaled to the channel dimensions, ensuring optimal coupling with the endothelial layer, while reducing parasitic resistance. As a result, TEER measurements can be performed over smaller sensing areas with higher signal stability and lower electrical noise [13]. Furthermore, such integration enables continuous and real-time TEER monitoring inside incubators, maintaining physiological temperature and flow conditions without disturbing the cellular microenvironment, an essential advancement toward accurate and standardized BBB quantification. All types of integrated electrodes applied in BBB-on-chip are classified in the following subsections.

#### 6.2.1. Array of Concentric Interdigitated Electrodes

Among integrated configurations, concentric interdigitated electrodes represent one of the most advanced geometries for TEER monitoring in BBB-on-chip systems. By alternating microscale anode and cathode fingers in a circular array, these electrodes establish a radially symmetric electric field and produce a homogeneous current distribution across the endothelial membrane [75]. This configuration minimizes edge effects and local current crowding, both of which often distort resistance measurements in linear or planar electrodes. In this design, electrodes are typically embedded within the upper and lower microfluidic channels, directly adjacent to the porous membrane, to ensure the sensing area corresponds precisely to the cultured BBB region [89]. The resulting alignment improves sensitivity while maintaining electrical isolation between channels. A representative example is the cyclic-olefin polymer-based BBB device [82], where a PC transparent membrane is sandwiched between two cyclic-olefin polymer layers with concentric interdigitated electrodes patterned on each side (Figure 5b). The Au electrodes are fabricated through sputter deposition on the polymer substrate, patterned using AZ^®^ 5214E photoresist lithography, and aligned to cover the full membrane area. The system enables impedance spectroscopy measurements across a broad frequency range (100 Hz–10 MHz), yielding approximately 2500 TEER readings. The resulting spectral data, analyzed via machine-learning algorithms, allows the automated classification of barrier states on the basis of subtle impedance variations; this approach demonstrates the potential of data-driven TEER analytics for real-time BBB assessment [82].

Despite these advantages, interdigitated electrode arrays require multiple photolithography steps, precise layer alignment, and surface treatment to ensure biocompatibility. Moreover, their high sensitivity to microchannel geometry and medium conductivity require careful calibration. Nevertheless, this design is a benchmark for next-generation and high-resolution TEER measurements, as it combines geometric uniformity with the analytical power of impedance spectroscopy and computational modeling, from which TEER can be robustly derived.

#### 6.2.2. Aluminum Sheet

A simpler, yet effective, integrated configuration was proposed by Duong et al. [83], who used aluminum sheet electrodes within a PDMS-based BBB-on-chip platform. In this model, a cellulose fiber membrane serves as the basement membrane, whereas hUVECs, human astrocytes, and brain pericytes are co-cultured under static conditions for seven days. Their system achieved a TEER value of 330 Ω.cm^2^, accompanied by >90% cell viability, demonstrating that the aluminum-based electrode could support stable barrier formation comparable to more expensive metallic systems.

The principal advantage of aluminum sheets lies in their low fabrication cost and ease of integration, as they can be patterned and embedded without clean-room facilities, making them attractive for rapid prototyping and scalable production [75]. Their large surface area also contributes to reduced contact resistance and improved measurement stability in static environments. However, aluminum’s high electrochemical reactivity in aqueous ionic media poses limitations for long-term use. The formation of an oxide layer (Al_2_O_3_) at the metal-electrolyte interface can alter impedance characteristics over time, subsequently introducing drift in TEER readings and potentially influencing local pH. However, passivation coatings or polymer encapsulation can address these problems. From a material perspective, aluminum electrodes represent a cost-effective entry point for integrated TEER measurements, suitable for proof-of-concept and short-duration studies. Yet for sustained, high-precision monitoring, particularly under dynamic flow, noble metals (e.g., Au, Pt) or conductive transparent oxides are preferred because of their chemical inertness and long-term stability.

#### 6.2.3. Deposited Electrodes

Deposited thin-film electrodes represent one of the most precise and reproducible strategies for TEER measurements in BBB-on-chip platforms. These electrodes consist of metallic films (commonly Au, Pt, chromium (Cr), or Ag) that are sputter-coated or evaporated onto rigid substrates such as glass or polymer layers. Subsequent masking or liftoff photolithography defines the electrode geometry with a micrometer-scale precision to enable a well-controlled shape, thickness, and transparency [10]. This fabrication approach allows direct quantification of the planar conductivity of endothelial monolayers while ensuring a uniform electric field distribution across the barrier. Booth et al. [43] provided a key comparative study using sputtered thin-film electrodes made of 150 nm Au, 20 nm Cr, and 800 nm Ag layers on glass substrates within a PDMS-based microfluidic BBB system. They observed a significant improvement in barrier tightness under dynamic flow; TEER values exceeded 250 Ω.cm^2^ for the microfluidic co-culture, compared to only 25 Ω.cm^2^ for the Transwell model. The uniform field generated by the deposited electrodes was a primary factor in this increase, as it minimized current distortion and edge effects. Martino et al. [84] further confirmed that Au thin-film electrodes provided enhanced stability and reproducibility for both static and dynamic conditions. Unlike traditional chopstick probes that require manual insertion, the integrated thin-film configuration permits real-time, noninvasive monitoring of barrier development during continuous culture. This feature enables a dynamic correlation between cellular maturation and electrical behavior, an essential capability for evaluating BBB response to drugs and stressors. Similarly, Palma-Florez et al. [67] engineered a PDMS-based chip with Au/Cr electrodes fabricated via photolithography and removal patterning on a glass substrate (Figure 5c). The close proximity of the electrodes to the endothelial zone ensured a homogeneous current density and reduced spatial variability in TEER measurements. Their multilayer process involved metal evaporation followed by a selective removal using an AZ^®^100 photoresist remover. This produced optically transparent, biocompatible electrodes compatible with live imaging.

Despite these advantages, deposited electrodes require complex and resource-intensive fabrication steps, including vacuum deposition, photolithography, and clean-room conditions [10]. Maintaining film adhesion on polymeric substrates and preventing metal ion leaching are additional challenges that demand specialized surface treatments. Nevertheless, the superior signal-to-noise ratio, long-term stability, and compatibility with real-time impedance spectroscopy make thin film-deposited electrodes an appropriate candidate for the high-fidelity electrical characterization of BBB-on-chip systems for obtaining accurate and reproducible TEER extraction.

#### 6.2.4. Screen-Printed Electrodes

Recent advances in 3D printed electronics have facilitated the fabrication of screen-printed electrodes as integrated sensing interfaces for BBB-on-chip platforms. In contrast to traditional vacuum-deposited films, screen-printed electrodes apply conductive pastes/inks (made of carbon, Pt, Au, or hybrid nanocomposites), which are printed directly on a flexible polymer substrate (such as PET, PDMS, or poly (methyl methacrylate) (PMMA)) [90,91,92,93]. These electrodes eliminate the need for clean-room facilities and significantly reduce cost and production time, which makes it attractive for scalable manufacturing and disposable chip designs. Moreover, the electrical and mechanical performance of printed electrodes can be modified by incorporating conductive nanofillers (such as graphene, carbon nanotubes, or metallic nanoparticles) into the polymer [94,95,96,97]. These composites combine mechanical flexibility with excellent conductivity and improve electrode-cell contact and ensure a stable operation during perfusion. The resulting conformal interface enhances signal uniformity, reduces interfacial impedance, and subsequently facilitates real-time TEER monitoring under dynamic flow conditions [89]. Krishnakumar et al. [85] developed a hybrid fabrication strategy that integrated screen printing and laser processing to produce a microfluidic platform with an embedded printed electrode array for real-time TEER monitoring. Their design minimized bubble formation, one of the most common issues in microfluidic measurements, and maintained excellent stability with only a 0.02 Ω variation in resistance after prolonged exposure to a cell culture medium. Importantly, the platform was low cost and disposable, overcoming scalability barriers that limit many microfabricated systems. This advance highlighted the promise of screen-printed electrodes for translational, clinically compatible organ-on-chip devices. In a complementary approach, Kawakita et al. [98] developed a carbon-based screen-printed electrode integrated into a PMMA-based BBB-on-chip system (Figure 5d). When hCMECs/D3 endothelial cells were exposed to increasing shear stress (from 0.05 to 2.7 dyne.cm^−2^), TEER values rose dramatically from 20 to 165 Ω.cm^2^. This result confirmed the physiological role of flow-induced tight junction reinforcement and validated the functional stability and sensitivity of carbon screen-printed electrodes under dynamic conditions.

Beyond cost efficiency and customizability, screen-printed electrodes are compatible with multiplexed TEER measurement arrays and enable the simultaneous analysis of multiple culture chambers or barrier zones. However, their performance is affected by ink components, layer thickness, and curing temperature, which can affect surface roughness, biocompatibility, and long-term signal drift. Optimization of surface coatings and ink formulations can become a key direction to improve the reproducibility and integrating of screen-printed electrodes into next generation BBB-on-chip designs.

#### 6.2.5. Dynamic External Electrode Configuration (Silver-Finger with ITO Design)

A novel advancement in BBB-on-chip electrical interfaces is the dynamic external electrode configuration, which integrates a movable fork-shaped Ag-coated electrode on the apical side and an ITO-coated glass substrate on the basal side (Figure 5e) [86]. This hybrid arrangement combines the high electrical conductivity of Ag with the optical transparency of ITO, enabling a simultaneous real-time TEER measurement, live-cell imaging, and controlled electroporation within the same microphysiological system. This design enables micron-scale positioning of the Ag-finger electrode relative to the endothelial monolayer, allowing precise control of the local electric-field intensity for consistent signal acquisition without causing electrical overstimulation. The ITO-coated basal glass serves as a counter-electrode, offering a stable, biocompatible interface with minimal optical interference, ideal for fluorescence-based barrier assays. Experimental runs produced TEER values ranging from 900 to 3500 Ω.cm^2^, with a stability over seven days in continuous culture.

Beyond TEER monitoring, this platform allows reversible electroporation of endothelial layers, enabling the controlled modulation of tight junctions to study drug permeability, gene delivery, and BBB repair. This multifunctional integration highlights its potential as a next generation electroactive BBB-on-chip system that combines sensing, stimulation, and therapeutic testing. However, several remaining engineering challenges include the precise calibration of electric-field gradients for avoiding the localized heating and the long-term stability of Ag coatings under repetitive electrochemical cycles. Future optimization may involve the replacement of Ag with inert conductive composites or introducing self-healing coatings to maintain performance under continuous operation.

**Table 3:** Structural and material characteristics of electrodes used for TEER measurements in BBB-on-chip systems.

Electrode Type	Material/Coating	Geometry/Design	Integration Location	Fabrication Method	Distinct Feature/Advantage	Ref.
Chopstick electrode	Pt, Ag/AgCl	Cylindrical wire pair	External inlet/outlet	Manual placement	Simple setup; compatible with standard TEER devices; ideal for Transwell models	[12,44,77,79]
Concentric interdigitated array	Au on cyclic-olefin polymer	Circular interdigitated rings	Top and bottom microchannels	Photolithography + sputter deposition	Homogeneous current field; suitable for impedance spectroscopy; high precision	[82,89,100]
Aluminum sheet electrode	Aluminum	Planar rectangular sheet	Integrated-side walls	Manual cutting and embedding	Low cost; eliminates the need for clean-room conditions; compatible with cellulose fiber membranes	[75,83]
Deposited thin-film electrode	Au/Cr or Au/Ag/Cr multilayer	Planar patterned film	Glass substrate/PDMS interface	Sputter coating + liftoff	High stability and real-time TEER monitoring; uniform field distribution	[10,43,67,84]
Screen-printed electrode	Carbon, Au, or Pt-based conductive ink	Flat printed pad	Integrated on polymer substrate	Screen printing/laser processing	Scalable; flexible; low cost; minimal signal drift (~0.02 Ω); suitable for dynamic flow	[85,89,98]
Dynamic Ag–ITO hybrid	Ag-coated fork + ITO glass	Movable fork (apical) + planar basal layer	External dynamic configuration	Physical coating + stepper motor positioning	Multifunctional: real-time TEER + reversible electroporation + live-cell imaging	[86]

## 7. Comparative Analysis of Electrode Architectures for TEER Measurements in BBB-on-Chip Systems

Electrode architecture is the primary determinant of TEER measurement fidelity, stability, and translational feasibility in BBB-on-chip systems. As evidenced by the systematic extraction summarized in Table 3 and Table 4, each electrode type embodies a balance between electrical performance, fabrication complexity, scalability, and long-term operational stability that ultimately dictate its suitability for quantitative and reproducible barrier assessments.

Chopstick electrodes represent the most available configuration, widely used because of their compatibility with standard TEER meters and are easily integrated into Transwell-like systems. However, their intrinsic nonuniform current distribution, high susceptibility to environmental noise, and their dependence on manual placement make them insufficient for precise microphysiological applications. These limitations create substantial variability and restrict their utility in standardized TEER evaluation. In contrast, integrated and deposited planar electrodes, including thin-film Au/Cr or Au/Ag/Cr multilayers, remain the benchmark for high-precision TEER assessments. Their patterned geometries support stable, homogeneous electric fields and enable real-time impedance monitoring with minimal drift. Nonetheless, their dependence on clean-room microfabrication, multi-step deposition workflows, and substrate-specific processing substantially limits accessibility and their adoption across research laboratories. Despite their superior performance, their high cost and low scalability limit their integration into high-throughput platforms. In this regard, screen-printed electrodes emerge as a compelling intermediate solution and offer a favorable combination of reproducibility, manufacturability, design flexibility, and exceptionally low signal drift (approx. 0.02 Ω). Their compatibility with polymeric substrates and suitability for dynamic-flow integration make them appropriate candidates for next-generation multichannel TEER modules. However, their deployment in microphysiological models still requires rigorous, long-term validation under physiologically relevant shear stress to confirm signal robustness, biointerface integrity, and an absence of electrode-induced cytotoxicity. Furthermore, hybrid configurations, which are exemplified by dynamic Ag-ITO systems, demonstrate the potential of multifunctional electrode assemblies capable of enabling tunable current profiles, reversible electroporation, and simultaneous TEER and imaging readouts. Although these systems achieve the highest reported TEER values (900–3500 Ω.cm^2^) and excellent stability over extended operations, they introduce new challenges, including corrosion risks, complex calibration requirements, and mechanical constraints associated with dynamic positioning. This comparative analysis shows how electrode performance parameters align with key biomimetic criteria required for physiologically relevant BBB-on-chip platforms.

Collectively, the evidence from Table 3 and Table 4 underscores that electrode architecture, rather than measurement protocol alone, governs the accuracy, reproducibility, and scalability of TEER quantification in BBB-on-chip systems. Integrated planar electrodes continue to deliver unmatched precision, whereas advances in printable conductive materials and hybrid composites are rapidly narrowing the performance gap between high-cost microfabricated devices and scalable low-cost alternatives. These technological developments are expected to catalyze the emergence of standardized, clinically translatable BBB-on-chip platforms capable of embedding real-time sensing, electrical stimulation, and multimodal functional readouts within a unified microphysiological system.

## 8. Challenges and Future Perspectives

The evolution from conventional 2D Transwell cultures to microfluidic BBB-on-chip platforms has enabled highly physiological relevance and real-time biosensing capabilities. Several commercial companies now provide ready-to-use BBB-on-chip kits that integrate biological and chemical modules for modeling the BBB [16]. Nevertheless, these systems remain limited in terms of standardization, scalability, and clinical translation.

A key limitation is the biological reproducibility of cell sources. Although human iPSCs have enabled developing personalized neurovascular models, their high cost, differentiation variability, and extended culture time limit their wide application. On the other hand, primary or immortalized brain endothelial cell lines offer better reproducibility and accessibility but do not fully replicate the human BBB phenotype and transport dynamics [101]. Therefore, hybrid co-culture models integrating iPSC-derived endothelium with primary astrocytes and pericytes may be a better approach for achieving physiological fidelity and experimental reproducibility.

From an engineering standpoint, TEER measurement and electrical sensing still lack standardization among laboratories. Variations in electrode geometry, current distribution, medium viscosity, and flow-induced shear stress introduce significant measurement inconsistencies for reported resistance values. Moreover, current TEER protocols often overlook frequency-dependent impedance spectra, which are essential for resolving the resistive and capacitive contributions of the barrier. The future of TEER-based BBB characterization therefore needs established standardized electrode architectures, reference calibration systems, and frequency-resolved electrical benchmarking frameworks that enable accurate and reproducible TEER derivation and true cross-laboratory comparability.

In terms of materials and device design, next generation BBB-on-chip platforms will likely integrate flexible electrodes, multielectrode array (MEA) biosensors, and nanostructured scaffolds to enable continuous, multimodal readouts. MEA-integrated TEER electrodes, recently applied to neural-on-chips, provide real-time electrophysiological feedback on cellular responses to drugs, toxins, or inflammatory stimuli. Moreover, electrospun nanofiber membranes with tunable porosity and mechanical properties can mimic the native basement membrane while supporting co-culture organization and directional signaling [102]. Hence, combining these technologies will obtain a multifunctional microphysiological platform that goes beyond passive modeling to active modulation and the monitoring of BBB behavior.

Considering the current rate of progression, it is expected that BBB-on-chip models will mimic real human BBB with much more accuracy. Additionally, real-time measurements will result in collecting a wide range of multiplex time series of data and independent information about the BBB. In this regard, machine-learning applications are proposed for analyzing these complex data sets, identifying hidden correlations, predicting barrier dysfunction, and optimizing culture parameters [103], as there will be a considerable amount of generated data sets of images and TEER values that provide rich sources for machine-learning algorithms. In this regard, developing data-driven digital BBB models could bridge experimental and computational modeling, accelerating predictive neuropharmacology.

The heterogeneity of reported TEER values across BBB-on-chip studies highlights the urgent need for standardized measurement and reporting protocols. By adopting the minimum information for reporting TEER checklist and electrode geometry, measurement settings, and calibration data, future studies can significantly improve interlaboratory reproducibility. Applying such standardized metadata reporting is expected to accelerate data harmonization, facilitate machine learning-based meta-analysis, and ultimately support acceptance of organ-on-chip models in neuropharmaceutical testing.

The future of BBB-on-chip research should combine standardized bioengineering, advanced electrode integration, and intelligent data analytics. Moreover, collaboration among material scientists, biologists, and computational modelers will be essential for transforming these microsystems from experimental tools into clinically translatable platforms for neurovascular research, drug discovery, and precision medicine.

## 9. Conclusions

BBB-on-chip technologies have transformed in vitro neurovascular modeling by enabling physiologically relevant shear stress, real-time biosensing, and multicellular co-culture design far beyond the capabilities of traditional static systems. This systematic review highlights that TEER, despite being the most established and sensitive assay for quantifying BBB integrity, remains highly dependent on electrode design and measurement conditions. Our comparative evaluation reveals that although deposited thin-film and interdigitated electrodes deliver high precision outputs and electrical stability, their fabrication complexity and reliance on clean-room lithography prevent scalability. In contrast, screen-printed and Ag-ITO hybrid configurations present advantages in manufacturability, cost efficiency, and compatibility with dynamic microfluidic environments, positioning them as strong candidates for future standardization. The design of bioinspired electrodes and standardization of TEER measurements are essential steps for developing BBB-on-chips that accurately mimic human physiological behavior.

The analysis highlights an urgent need for harmonized TEER reporting practices in regard to electrode geometry, frequency-dependent electrical impedance behavior, environmental control, and blank resistance correction. Adoption of the proposed metadata framework would significantly reduce interlaboratory variability and enable more meaningful cross-platform comparisons. Looking forward, the merging of advanced electrode integration, flexible microelectronics, machine learning-driven impedance analytics, and biomimetic scaffold engineering is expected to redefine BBB-on-chip systems as strong and predictive tools for CNS drug development. The collaboration of engineers, biologists, and computational scientists will be essential for transitioning these platforms from research prototypes toward standardized, industry-ready microphysiological systems.

Despite significant advances in TEER electrode integration for BBB-on-chip systems, several challenges remain. The long-term electrical and electrochemical stability of electrodes under continuous perfusion and physiologically relevant shear stress remains a critical limitation for chronic barrier monitoring. Moreover, translating high-performance electrode architectures to scalable, high-throughput platforms suitable for drug screening requires fabrication strategies to balance precision, cost, and reproducibility. Broader adoption of BBB-on-chips will ultimately depend on establishing standardized reporting frameworks and aligning with emerging regulatory expectations to enable interlaboratory comparability and translational validation.

## Figures and Tables

**Table 1:** Quality assessment criteria and scoring framework (maximum score 20).

Criterion	Score Range
Electrode accuracy	0–4
Long-term stability	0–4
Scalability	0–4
Fabrication cost	0–3
Cleanroom dependency	0–3
Suitability for dynamic microfluidic conditions	0–2

**Table 4:** Comparative performance, scalability, and functional evaluation of TEER electrodes.

Electrode Type	Flow Condition	Measured TEER (Ω.cm^2^)	Accuracy/Signal Stability	Scalability	Cost	Long-Term Stability	Limitations/Remarks
Chopstick	Static/limited dynamic	30–200	Low; sensitive to noise	High (easy use)	Low	Moderate (requires heating control)	Nonuniform current; unsuitable for high-precision TEER
Concentric interdigitated array	Dynamic	Up to 300	High; uniform current profile	Medium	High	High	Complex fabrication; requires clean-room setup
Aluminum sheet	Static	Approx. 330 ± 4.2	Moderate	High	Very low	Good (7 days)	Limited transparency’ static only
Deposited thin film	Static/dynamic	250–1000	Very high; stable impedance	Low (clean-room limited)	High	Excellent	High cost, limited scalability
Screen-printed	Static/dynamic	20–165	High (minimal drift 0.02 Ω)	Very high	Very low	Good (culture stable)	Requires validation under prolonged flow
Ag-ITO hybrid (dynamic)	Dynamic	900–3500	Excellent, tunable field control	Medium	Moderate	High (>7 days)	Requires precise calibration; Ag corrosion risk

## Data Availability

All data supporting this study are included in the main text and its tables.

## Abbreviations

The following abbreviations are used in this manuscript:

BBB	Blood-brain barrier
TEER	Transendothelial electrical resistance
CNS	Central nervous system
iPSC	Induced pluripotent stem cell
ECs	Endothelial cells
ZO	Zonula occludens
HES	Hydroxyethyl starch
PET	Polyethylene terephthalate
PC	Polycarbonate
PDMS	Polydimethylsiloxane
hCMECs	Human cardiac microvascular endothelial cells
hBMECs	Human brain microvascular endothelial cells
BMVECs	Brain microvascular endothelial cells
hUVECs	Human umbilical vein endothelial cells
hBVPs	Human brain vascular pericytes
FITC	Fluorescein isothiocyanate
Pt	Platinum
Ag/AgCl	Silver/silver chloride
Au	Gold
ITO	Indium tin oxide
LY	Lucifer yellow
EBA	Evans blue-albumin
cerebEND	Cerebellar capillary endothelial cells
Cr	Chromium
Ag	Silver
PMMA	Methyl methacrylate
R_TEER_	Ohmic resistance of the cell layer
R_M_	Cell culture medium
R_I_	Porous membrane
R_EMI_	Electrode-medium interface
CAD	Computer-aided design
MEA	Multielectrode array
